# Adverse respiratory outcomes among patients with resolved asthma: matched retrospective cohort study

**DOI:** 10.3399/BJGP.2023.0271

**Published:** 2024-04-30

**Authors:** Hywel Jones, Bethan Cumins, Rebecca Cannings-John, Haroon Ahmed

**Affiliations:** Division of Population Medicine, Cardiff University, Cardiff.; Health Education and Improvement Wales, Nantgarw.; Centre for Trials Research, Cardiff University, Cardiff.; Division of Population Medicine, Cardiff University, Cardiff.

**Keywords:** asthma, general practice, respiratory tract infections

## Abstract

**Background:**

Patients with asthma may have symptom remission leading to a primary care code of resolved asthma. Little is known about subsequent rates of exacerbations and respiratory tract infections (RTIs).

**Aim:**

To assess the risk of adverse respiratory outcomes for people with resolved asthma compared with those with active asthma and without asthma.

**Design and setting:**

This was a retrospective cohort study of patients aged >5 years, registered with a general practice in England contributing data to the Clinical Practice Research Datalink between January 2010 and December 2019.

**Method:**

Patients with resolved asthma were matched to non-asthma controls and active asthma controls for age, sex, and practice. Negative binomial regression was used to estimate incidence rate ratios (IRRs) with 95% confidence intervals (CIs) for asthma exacerbations, RTIs, flu/pneumonia, and antibiotic prescriptions.

**Results:**

Cohorts included 16 023 patients (8720 (54.4%) females, mean age 37 years). Compared with the active asthma cohort, the resolved cohort had fewer hospital admissions (adjusted IRR 0.29, 95% CI = 0.27 to 0.32) and general practice consultations (adjusted IRR 0.05, 95% CI = 0.04 to 0.07) for asthma exacerbations. The resolved and non-asthma cohorts had similar rates of hospital admissions for RTIs or flu/pneumonia. However, the resolved cohort had significantly greater incidence of general practice consultations for lower RTIs (adjusted IRR 2.34, 95% CI = 2.08 to 2.64) and antibiotic prescriptions (adjusted IRR 1.37, 95% CI = 1.30 to 1.44).

**Conclusion:**

Patients with resolved asthma had greater risk of general practice RTI and antibiotic prescription than the general population and may benefit from defined strategies for reassessing symptoms and reinitiating asthma therapy.

## Introduction

Asthma is a chronic respiratory condition affecting around 339 million people worldwide.^1^ Asthma is characterised by variable airflow limitation, bronchial hyperresponsiveness, mucus hypersecretion, and airway inflammation leading to airway narrowing that causes symptoms of wheeze, breathlessness, and chest tightness for people with the disease.^2^

A UK study found that the lifetime prevalence of patient-reported clinician-diagnosed asthma was 15.6% but the annual prevalence of patient-reported clinician-diagnosed symptomatic asthma was 8.1%, suggesting a proportion of people will have a period of no symptoms following their asthma diagnosis, that is, a period of remission.^3^ Remission is more likely in those with childhood-onset asthma and estimates range from 48–60% for onset before the age of 10 years to 5–15% for onset after the age of 20 years.^4^^–^^6^ However, people with asthma in remission still have evidence of bronchial hyperresponsiveness, significant reversibility, worse lung function, ongoing airway inflammation, and significantly greater levels of eosinophilia, when compared with non-asthma controls.^7^ Estimates for relapse among people with remission of asthma range from 19–44%.^8^^,^^9^

In England and Wales, long-term management of asthma is primarily undertaken by general practice. In 2004, key performance indicators were set by the Quality and Outcomes Framework (QOF) and included maintenance of a register of patients with asthma, and attainment of the following measures in a defined proportion of those on the register:

- measures of variability or reversibility recorded;
- an asthma review in the preceding 12 months; and
- a record of smoking status in the preceding 12 months.^10^

The framework recognised that asthma symptoms can remit and stated it was inappropriate to monitor symptom-free patients on no therapy or very occasional therapy. They requested that the asthma register was constructed annually by searching for patients with a history of asthma but excluding those with remission, defined as those with no asthma medication prescriptions in the prior 12 months.^11^ Diagnostic codes for ‘resolved asthma’ were also introduced for use in the general practice electronic health record.

**Table:** How this fits in

Patients with asthma may have a period of remission and be regarded as having ‘resolved asthma’ in their primary care record. Despite resolution of symptoms, patients with resolved asthma may have ongoing respiratory pathophysiology and little is known about their ongoing risk of adverse respiratory events. This study found that patients in UK primary care with a record of resolved asthma have a greater risk of respiratory tract infection and antibiotic use than the general population, but one that is lower than patients with active asthma. Patients with resolved asthma may benefit from a comprehensive respiratory assessment if they present with symptoms of a respiratory tract infection to assess symptom burden, airway obstruction, and the potential value of reinitiating inhaled treatment.

Despite evidence for ongoing respiratory pathophysiology and relatively high rates of relapse, there is no guidance for regular assessment or review of people deemed to have resolved asthma, and little is known about their ongoing risk of adverse respiratory events. Therefore, the aim of this study was to assess the risk of asthma exacerbations, respiratory tract infections (RTIs), and antibiotic prescribing for people with a diagnostic code of ‘resolved asthma’ compared with those with active asthma and matched non-asthma controls.

## Method

### Data source

This study used anonymised longitudinal general practice data from the GOLD version of the UK Clinical Practice Research Datalink (CPRD).^12^ Practices contributing data to CPRD GOLD are audited to assess the reliability and accuracy of data recording. Patient-level data are considered ‘acceptable’ for inclusion in the CPRD if internally consistent in recording of age, sex, registration details, and clinical events. The CPRD GOLD sample represents 4.6% of the UK population and 4.9% of UK general practice^1^ and is broadly representative of the wider UK population in terms of age and sex distribution.^13^ Practices ‘opt in’ to contribute data to CPRD and about 50% of practices contributing to CPRD GOLD provide additional consent to allow linkage of patient-level data with other datasets, including hospital admission data.^14^

The study protocol was approved by the Independent Scientific Advisory Committee for CPRD on 6 September 2019 (protocol ref: 19-187).

### Study design, population, and follow-up

This was a retrospective cohort study of patients alive and registered with a CPRD practice between January 2010 and December 2019. Patients were eligible for inclusion if they were aged >5 years, their data were assessed by CPRD to be of sufficient quality, their registered practice was ‘up to standard’, they had more than 1 day of follow-up in the CPRD, and they were eligible for linkage with hospital admission data.

Three cohorts were constructed:

- active asthma cohort;
- resolved asthma cohort; and
- non-asthma cohort.

#### Active asthma cohort

These were patients with an asthma-specific diagnostic code (Supplementary Table S1) at any point in their GP record and at least one prescription for asthma medication between 1 January 2010 and 31 December 2019. Asthma-specific diagnostic codes have a positive predictive value of 86.4% for identifying someone with asthma.^15^ Patients with a resolved asthma Read code at any point in their GP record were excluded. This cohort were followed from the date of their first prescription for asthma medication during the study period (index date), to the earliest of date where no asthma medications were prescribed in the prior 12 months (that is, the date they would meet the definition of resolved asthma), the end of the study period (31 December 2019), date of transfer out of the practice, or date of death.

#### Resolved asthma cohort

These were patients with at least one code for ‘resolved asthma’ (Supplementary Table S2) between 1 January 2010 and 31 December 2019. This cohort were followed from the date of their first resolved asthma Read code during the study period (index date), to the earliest date where an asthma medication was prescribed (that is, the date they no longer met the definition of resolved asthma), the end of the study period, date of transfer out of the practice, or date of death.

Both the resolved asthma cohort and the active asthma cohorts were restricted to those aged >5 and <90 years on the index date, with 365 days of ‘look-back’ data before the index date to allow ascertainment of relevant sociodemographic and clinical characteristics, and enable a sensitivity analysis with stricter definitions of resolved and active asthma. Those with a chronic obstructive pulmonary disease (COPD)-related Read code (Supplementary Table S3) were excluded to avoid misclassification related to asthma–COPD overlap or diagnostic uncertainty.

Participants with periods of both resolved and active asthma only contributed to the resolved asthma cohort, given that their periods of active asthma likely differ from those without a resolved asthma period.

The resolved asthma and active asthma cohorts were matched one-to-one using nearest neighbour matching without replacement. Matching variables were practice, sex (exact matching), and age at index date (1 year either way). Matching was undertaken with R package Matchit version 4.4.0.^16^

#### Non-asthma cohort

CPRD provided a population-based cohort of patients without active or resolved asthma or COPD in the same study period, eligible for linkage to hospital admission data, with at least 1 day of follow-up during the study period. CPRD used index date matching. In this algorithm, the ‘case’ patient (here, the patient with resolved asthma) has a specified index date that must fall between the follow-up start and follow-up end dates of the ‘control’ patient. Non-asthma patients were matched one-to-one with the resolved cohort on practice, sex, and year of birth. The index data for non-asthma controls were set to be that of the corresponding case patient. Patients aged >5 and <90 years on the index date were excluded.

### Outcomes

The primary outcomes were:

- the number of hospital admissions for asthma (comparing resolved asthma with active asthma); and
- the number of hospital admissions for upper RTIs (URTIs) and lower RTIs (LRTIs), flu, and pneumonia (comparing resolved asthma with non-asthma controls, and active asthma with non-asthma controls).

Secondary outcomes were general practice consultations for asthma exacerbations (comparing resolved asthma with active asthma), LRTI, and all-cause antibiotic prescriptions (comparing resolved asthma with non-asthma controls, and active asthma with non-asthma controls).

### Statistical analysis

The sociodemographic and clinical characteristics of each cohort, including details of QOF-related asthma indicators, are described. The numbers of each outcome that occurred during the relevant person-time were calculated. To allow for overdispersion, negative binomial regression models, rather than Poisson models, were used to estimate incidence rate ratios (IRRs) and 95% confidence intervals (CIs) for each outcome among the resolved and active asthma cohorts compared with the non-asthma cohort. Cohorts were already matched for age, sex, and practice. Using a mixed-effects model with practice as a random intercept effect, the models were also adjusted for age and sex (because of some imbalance, despite matching), as well as for Charlson comorbidity score, Index of Multiple Deprivation (IMD) quintile (quintile 1 being most deprived and 5 least deprived), and smoking status (non-smoker, ex-smoker, current smoker) with log of length of follow-up period as an offset.

Smoking status was identified from the most recent relevant codes recorded in the general practice data before the index date. IMD was based on the patient’s postcode, linked to lower layer super output areas. The Charlson comorbidity score was based on all the relevant clinical codes found on or before the index date. A small number of people with missing IMD data were excluded (three in the active asthma cohort, six in the resolved asthma cohort, and eight in the non-asthma cohort). Missing smoking status data (1561 [9.7%] in the resolved asthma cohort, 1266 [7.9%] in the active asthma cohort, and 3533 [22.0%] of the non-asthma cohort) were imputed using multiple imputation with chained equations, implemented in the ‘mice’ package in R.^17^ Smoking was modelled as an unordered categorical variable using the Bayesian polytomous regression model and the other following variables: cohort, region, sex, year of birth, Charlson score, years of follow-up, and all relevant outcome indicators. Ten imputations with 10 iterations were used. Negative binomial regression models were run on each of the 10 imputed datasets and estimates were combined using Rubin’s rule.

In sensitivity analyses, to apply stricter definitions of resolved and active asthma, the asthma cohort was restricted to those with at least one prescription of an asthma medication in the 12 months before the index date, and the resolved cohort was restricted to those without any prescription of an asthma medication in the 12 months before the index date.

Analyses were undertaken in R version 4.2.2 and reported according to RECORD guidelines.^18^

## Results

**Figure 1.**
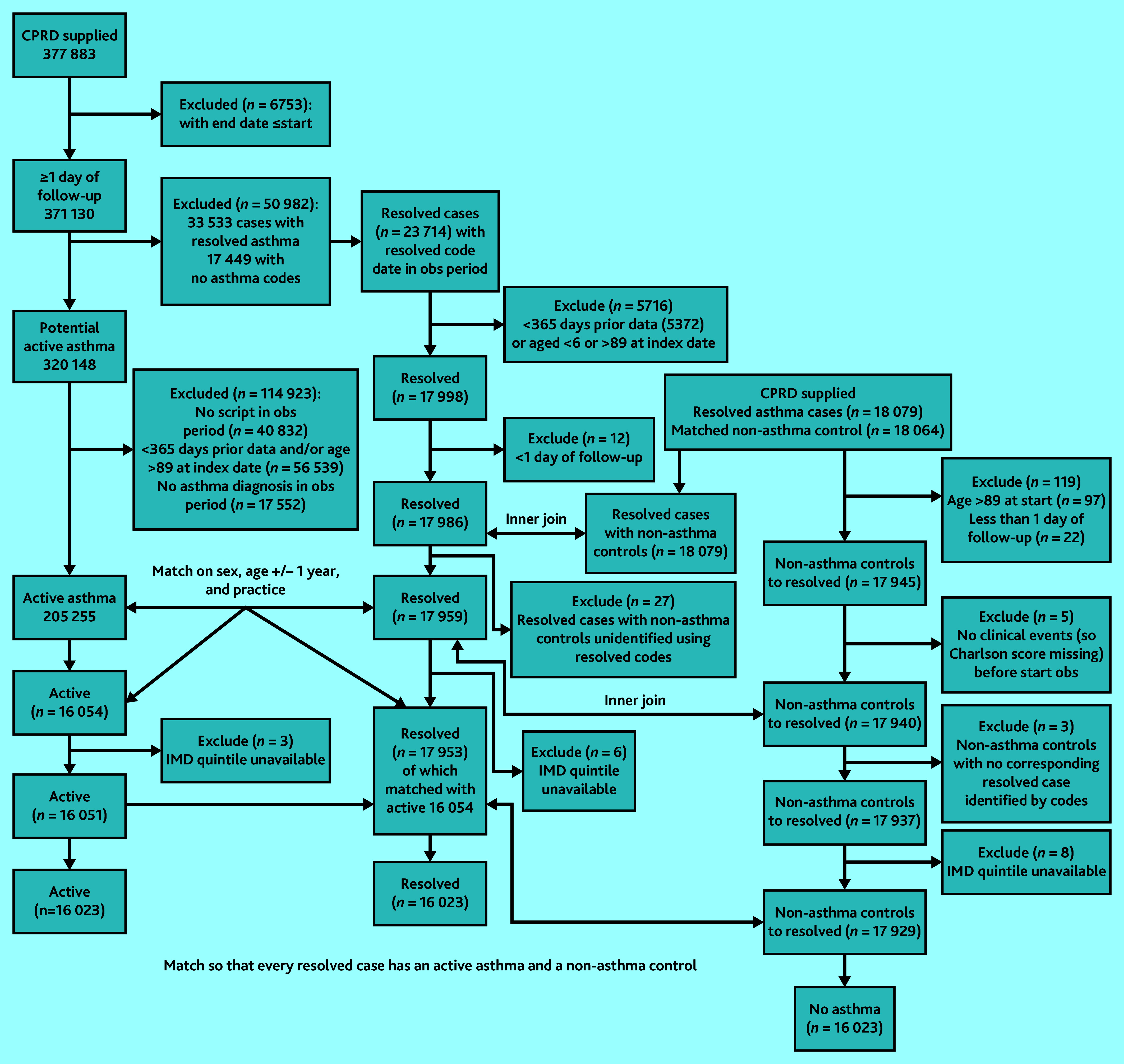
Flow of patients into each cohort. IMD = Index of Multiple Deprivation. Obs = observation.

Figure 1 shows details of the flow of patients into each cohort. After exclusions and 1:1:1 matching on age, sex, and practice, 16 023 patients remained in each cohort, with 8720 (54.4%) being female (Table 1). Mean ages at the start of follow-up across the matched cohorts were 37.1 (standard deviation [SD] 20.3) years in the resolved asthma cohort, 37.4 (SD 20.2) years in the active asthma cohort, and 37.1 (SD 20.3) years in the non-asthma cohort.

**Table 1.** Descriptive characteristics of the three cohorts

**Characteristic**	**Resolved asthma (*n* = 16 023)**	**Active asthma (*n* = 16 023)**	**Non-asthma (*n* = 16 023)**
**Sex**			
Male	7303 (45.6)	7303 (45.6)	7303 (45.6)
Female	8720 (54.4)	8720 (54.4)	8720 (54.4)

**Age at start FUP, years, mean (SD)**	37.06 (20.27)	37.41 (20.24)	37.06 (20.27)

**Years of follow-up, mean (SD)**	1.99 (1.86)	3.54 (2.58)	2.68 (2.02)

**Index of Multiple**			
**Deprivation quintile**			
1 — most deprived	3854 (24.1)	3695 (23.1)	3849 (24.0)
2	3583 (22.4)	3514 (21.9)	3570 (22.3)
3	3134 (19.6)	3158 (19.7)	3061 (19.1)
4	2876 (17.9)	2961 (18.5)	2893 (18.1)
5 — least deprived	2576 (16.1)	2695 (16.8)	2650 (16.5)

**Charlson score^a^**			
0	680 (4.2)	531 (3.3)	14 249 (88.9)
1	13 425 (83.8)	13 582 (84.8)	770 (4.8)
2	744 (4.6)	728 (4.5)	677 (4.2)
3	752 (4.7)	772 (4.8)	168 (1.0)
4	225 (1.4)	223 (1.4)	92 (0.6)
≥5	197 (1.2)	187 (1.2)	67 (0.4)

**Smoking status**			
Non-smoker	9198 (57.4)	9167 (57.2)	7618 (47.5)
Ex-smoker	2528 (15.8)	2726 (17.0)	2128 (13.3)
Current smoker	2736 (17.1)	2864 (17.9)	2744 (17.1)
Missing	1561 (9.7)	1266 (7.9)	3533 (22.0)

**Spirometry recorded in observation period, yes**	880 (5.5)	310 (1.9)	—

**FEV_1_ recorded in observation period, yes**	910 (5.7)	374 (2.3)	—

**Annual asthma review recorded in observation period, yes**	3658 (22.8)	11 779 (73.5)	—

a *Charlson score of 0 indicates no comorbidity. Values are* n *(%) unless otherwise indicated. FEV_1_ = forced expiratory volume in 1 second. FUP = follow-up. SD = standard deviation.*

Charlson scores and smoking status were similar between the resolved and active asthma cohorts but the non-asthma cohort had a substantially greater proportion of people with no comorbidity (a Charlson score of 0), and with missing smoking status. Compared with the active asthma cohort, greater proportions of the resolved asthma cohort had spirometry and forced expiratory volume in 1 second recorded before the index date, but fewer had an asthma review recorded in the 15 months before the index date.

Compared with the active asthma cohort, the resolved asthma cohort had fewer asthma hospital admissions (18.28 versus 4.61 per 100 person-years, adjusted IRR 0.29, 95% CI = 0.27 to 0.32) (Table 2). Compared with the non-asthma cohort, the active asthma cohort had significantly greater hospital admissions for LRTI (0.15 versus 0.72 per 100 person-years, adjusted IRR 3.04, 95% CI = 2.22 to 4.18) and flu/pneumonia (0.29 versus 0.78 per 100 person-years, adjusted IRR 1.80, 95% CI = 1.40 to 2.31). There were no significant differences between the non-asthma and resolved asthma cohort for hospital admissions for URTI, LRTI, or flu/pneumonia.

**Table 2.** IRR and 95% CIs for primary and secondary outcomes

**Outcome and cohort**	**Events**	**Person-time, years**	**Crude IRR (95% CI)^a^**	**Adjusted IRR (95% CI)^b^**
**Asthma hospital admissions**				
Active asthma	9904	54 179	Reference	Reference
Resolved asthma	1472	31 965	0.28 (0.26 to 0.31)	0.29 (0.27 to 0.32)

**URTI hospital admissions**				
Non-asthma	63	42 969	Reference	Reference
Active asthma	138	56 741	1.65 (1.19 to 2.30)	0.92 (0.62 to 1.37)
Resolved asthma	63	31 965	1.36 (0.92 to 1.99)	0.89 (0.55 to 1.44)

**LRTI hospital admissions**				
Non-asthma	66	42 969	Reference	Reference
Active asthma	410	56 741	4.52 (3.37 to 6.05)	3.04 (2.22 to 4.18)
Resolved asthma	82	31 965	1.82 (1.22 to 2.72)	1.30 (0.87 to 1.95)

**Flu or pneumonia hospital**				
**admission**				
Non-asthma	123	42 969	Reference	Reference
Active asthma	443	56 741	2.70 (2.13 to 3.41)	1.80 (1.40 to 2.31)
Resolved asthma	122	31 965	1.39 (1.02 to 1.90)	1.08 (0.78 to 1.50)

**GP asthma exacerbation consultations**				
Active asthma	3816	56 741	Reference	Reference
Resolved asthma	108	31 965	0.05 (0.04 to 0.07)	0.05 (0.04 to 0.07)

**GP LRTI consultations**				
Non-asthma	804	42 969	Reference	Reference
Active asthma	6985	56 741	6.19 (5.68 to 6.74)	5.27 (4.79 to 5.78)
Resolved asthma	1489	31 965	2.87 (2.57 to 3.19)	2.34 (2.08 to 2.64)

**GP all-cause antibiotic prescriptions**				
Non-asthma	17 285	42 969	Reference	Reference
Active asthma	63 222	56 741	2.64 (2.54 to 2.73)	2.08 (1.99 to 2.17)
Resolved asthma	20 543	31 965	1.83 (1.75 to 1.91)	1.37 (1.30 to 1.44)

a *Unadjusted models matched on age and sex with the practice included as a random effect to account for clustering.*

b *Models adjusted for age, sex, Index of Multiple Deprivation, Charlson score, and smoking status (imputed where missing), with the practice included as a random effect. CI = confidence interval. IRR = incidence rate ratio. LRTI = lower respiratory tract infection. URTI = upper respiratory tract infection.*

Compared with the active asthma cohort, the resolved asthma cohort had fewer general practice consultations for asthma exacerbation (6.73 versus 0.34 per 100 person-years, adjusted IRR 0.05, 95% CI = 0.04 to 0.07) (Table 2). Compared with the non-asthma cohort, the active and resolved asthma cohorts had significantly greater general practice consultations for LRTI (non-asthma versus active asthma cohort, 1.87 versus 12.31 per 100 person-years, adjusted IRR 5.27, 95% CI = 4.79 to 5.78; non-asthma versus resolved asthma cohort, 1.87 versus 4.66 per 100 person-years, adjusted IRR 2.34, 95% CI = 2.08 to 2.64). Active and resolved asthma cohorts also had greater all-cause antibiotic prescriptions (non-asthma versus active asthma cohort, 40.22 versus 111.42 per 100 person-years, adjusted IRR 2.08, 95% CI = 1.99 to 2.17; non-asthma versus resolved asthma cohort, 40.22 versus 64.27 per 100 person-years, adjusted IRR 1.37, 95% CI = 1.30 to 1.44).

In sensitivity analyses, 3105 (19.4%) patients were excluded from the active asthma cohort (no asthma medication in the 12 months before index date) and 5333 (33.3%) from the resolved cohort (at least one asthma medication in the 12 months before the index date). There were no appreciable differences for the findings (Table 3).

**Table 3.** IRR and 95% CIs for primary and secondary outcomes using stricter definitions of resolved and active asthma

**Outcome, cohort**	**Adjusted IRR (95% CI)^a^**
**Asthma hospital admissions**	
Active asthma	Reference
Resolved asthma	0.25 (0.23 to 0.28)

**URTI hospital admissions**	
Non-asthma	Reference
Active asthma	0.69 (0.43 to 1.11)
Resolved asthma	0.60 (0.31 to 1.15)

**LRTI hospital admissions**	
Non-asthma	Reference
Active asthma	3.20 (2.30 to 4.46)
Resolved asthma	1.06 (0.66 to 1.71)

**Flu or pneumonia hospital admission**	
Non-asthma	Reference
Active asthma	1.83 (1.42 to 2.37)
Resolved asthma	1.09 (0.75 to 1.58)

**GP asthma exacerbation consultations**	
Active asthma	Reference
Resolved asthma	0.04 (0.03 to 0.06)

**GP LRTI consultations**	
Non-asthma	Reference
Active asthma	5.39 (4.87 to 5.96)
Resolved asthma	2.09 (1.81 to 2.40)

**GP all-cause antibiotic prescriptions**	
Non-asthma	Reference
Active asthma	2.20 (2.10 to 2.30)
Resolved asthma	1.21 (1.13 to 1.28)

a *Models adjusted for age, sex, Index of Multiple Deprivation, Charlson score, and smoking status (using complete cases only), with the practice as a random effect. CI = confidence interval. IRR = incidence rate ratio. LRTI = lower respiratory tract infection. URTI = upper respiratory tract infection.*

## Discussion

### Summary

In this matched retrospective cohort study, respiratory outcomes were compared among patients with active asthma, resolved asthma, and non-asthma controls. Patients with resolved asthma had significantly fewer hospital and general practice-attended asthma exacerbations than those with active asthma. Compared with non-asthma controls, patients with resolved asthma had similar rates of hospital admission for URTI, LRTI, and flu/pneumonia, but greater incidence of general practice-attended LRTIs and all-cause antibiotic prescriptions. These differences remained similar when a stricter definition of resolved asthma was applied.

### Strengths and limitations

This study used a large representative sample of patients with active, and resolved asthma, and matched non-asthma controls. Respiratory events were determined from health records rather than self-report making them less prone to bias from recall or diagnostic misclassification. Coding of asthma in general practice records is relatively accurate and reliable.^15^ Sensitivity analyses were undertaken with stricter definitions of active and resolved asthma using data on prescribed medications that are well recorded in general practice records.

Usage and interpretation of the resolved asthma Read codes are likely to vary between clinicians and practices. As resolved asthma required a period of >12 months without any asthma-related medications it is likely that this is a heterogeneous group with resolution of symptoms for different periods of time before the code being recorded. Compared with the non-asthma cohort, the relatively greater number of comorbidities in the resolved asthma cohort may have led to ascertainment bias of the respiratory outcomes studied, where respiratory symptoms were discussed during a hospital admission or a general practice consultation for other problems.

### Comparison with existing literature

The finding in this study of greater risk of respiratory adverse outcomes among people with active asthma compared with non-asthma is unsurprising and aligns with survey research from across the Nordic countries that found people with asthma reported substantially higher rates of LRTIs and antibiotic use than people without.^19^ Rates of asthma exacerbation among the active asthma cohort in the current study were similar to previous studies from the CPRD.^20^

To the best of the authors’ knowledge, no estimates exist for rates of asthma exacerbation or respiratory infection among a resolved asthma cohort. The current study found that people with resolved asthma had greater incidence of general practice-attended LRTIs and all-cause antibiotic prescriptions than people without asthma. This supports existing evidence for ongoing airway hyperresponsiveness, inflammation, and remodelling with varying degrees of airway obstruction, among people with a period of asthma remission.^7^^,^^21^ It highlights that, in some people, symptom report may be insufficient to determine that asthma is in true remission^7^ and supports debate around the importance of recognising clinical (absence of asthma symptoms, exacerbations, and treatment) versus complete remission (clinical remission plus evidence of absence or normalisation of underlying pathology including inflammation).^22^ Patients in the current study in the resolved asthma cohort likely reflect clinical remission, and recurrence of asthma symptoms may be recorded as non-specific LRTIs rather than asthma exacerbations.

### Implications for research and practice

The key clinical implication of this research is that patients in UK primary care with a record of resolved asthma have a greater risk of LRTI and antibiotic use than the general population, but one that is lower than patients with active asthma. They are therefore a currently less clear ‘middle group’ with no guidance around formal regular review of respiratory symptoms. The increased rate of LRTIs may represent asthma relapse and some of these might be preventable if inhaled asthma medication were reinitiated. A pragmatic approach may be to undertake a more comprehensive respiratory assessment if a patient with resolved asthma presents with symptoms of LRTI, to assess symptom burden, airway obstruction, and the potential value of inhaled treatment.

Future research should aim to better quantify rates of remission and relapse among people with asthma, and identify clinically useful and acceptable predictors of both, preferably usable in primary care, to aid decisions about stopping and re-starting asthma treatments.

## Data

CPRD data agreement prevents data sharing. Researchers can apply directly to CPRD for a dataset.
